# Identification of Pen m 4 as a potential cause of occupational asthma to Gammarus shrimp

**DOI:** 10.1186/s13601-018-0232-9

**Published:** 2018-11-09

**Authors:** A. Sogo, M. J. Cruz, M. J. Amengual, X. Muñoz

**Affiliations:** 1 Pulmonology Department, Corporacio Sanitaria Parc Tauli, Sabadell, Spain; 2 Pulmonology Department, Servei de Pneumologia, Hospital General Vall d’Hebron, Passeig Vall d’Hebron, 119, 08035 Barcelona, Spain; 3 CIBER Enfermedades Respiratorias (Ciberes), Barcelona, Spain; 4 Immunology Department, Corporacio Sanitaria Parc Tauli, Sabadell, Spain; 5 Department of Cell Biology, Physiology and Immunology, Universidad Autónoma de Barcelona, Barcelona, Spain

**Keywords:** Specific bronchial provocation test, IgE, Tropomyosin, Allergy

## Abstract

We present the case of a 34-year-old male patient employed for 8 years in a company manufacturing and packaging animal feed. The patient developed occupational asthma to dry Gammarus powder. The diagnosis was confirmed by specific bronchial provocation test. The determination of specific IgE antibodies was positive for Pen m 4, a sarcoplasmic calcium binding protein, with a level of 6.7 ISU-E. The sensitization to Pen m 4 described here may identify a new allergen causing occupational asthma in these workers.

## To the Editor,

Environmental exposure to seafood dust, smoke or vapors is a known cause of occupational asthma (OA), with a prevalence between 2 and 36% [1, 2]. The differences in prevalence are partly due to the type of seafood processing and handling. Asthma associated with exposure to dry shrimp powder is an entity that has been poorly documented [2–5]. Isolated cases of OA have been described after domestic exposure to dry turtle food [3, 4] and in workers at fish-food factories [4, 5]. Most affected workers are commonly sensitized to more than one allergen, and sometimes these allergens cross-react with other shellfish, mite and cockroach allergens [3–6].

We present the first case of a patient who developed OA due to exposure to shrimp Gammarus powder and with specific sensitization to Pen m 4. This is a sarcoplasmic calcium binding protein (SCBP) which may present cross-reactivity with other crustacean species, but to our knowledge has not been shown to cross-react with mites or cockroaches [7].

The patient was a 34-year-old male with no previous history of allergic disease and no toxic habits who had worked for 8 years in a company manufacturing and packaging animal feed (among them shrimp Gammarus for turtle and fish feed). His main activity during the workday was mixing and packing the feed and he was responsible for the supervision of the packaging of these foods. After 2 years of exposure, he began to develop episodes of rhinorrhea, dyspnea, cough and wheezing which were directly related to the environmental exposure to dry shrimp Gammarus powder. The symptoms appeared regularly and with great intensity on the day of the week in which the shrimp Gammarus was packed. The patient reported improvement during the weekend and vacation periods. 6 months after the onset of respiratory symptoms he began to present oral pruritus immediately after eating shrimps, and so he removed them from his diet.

In the initial evaluation, a first blood test showed 11% eosinophils and a total IgE of 145 kU/L. Specific IgE and prick tests to common pneumoallergens (mites, pollens, fungi, epithelia and latex), panallergens (tropomyosin, profilin, polcalcin and LTP), flours (rye, corn, wheat, soy, oats), mollusks and fish, were negative. The patient only showed a positive cutaneous reaction of 12 × 12 mm to shrimp extract, and 10 × 10 mm in the prick by prick to shrimp Gammarus. A specific IgE against shrimp was positive, at a value of 1.54 kU/L. Spirometry showed a forced vital capacity (FVC) of 104%, a forced expiratory volume in the first second (FEV1) of 89% and a FEV1/FVC ratio of 68%, with a positive bronchodilator test (PBD) of 20%. The chest radiograph was normal.

**Fig. 1:**
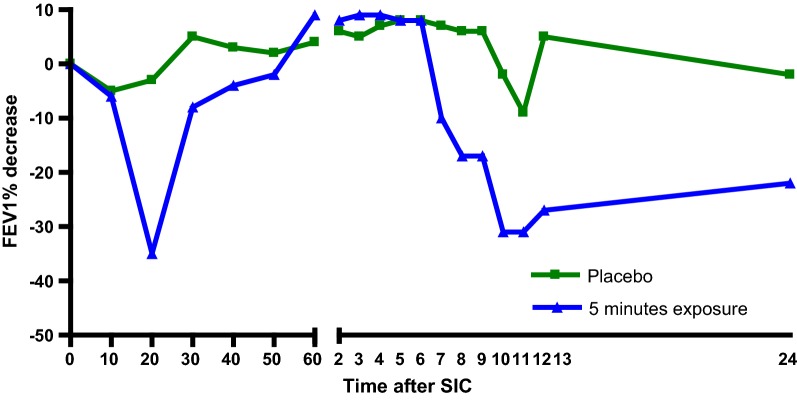
Result of specific inhalation challenge to Gammarus dust

Given the suspicion of OA, a specific inhalation challenge was carried out by tipping 50 g of shrimp Gammarus dust mixed in 100 g of lactose from one tray to another at a distance of 30 cm from the nose for 5 min, as previously described [8]. The test was positive, with the patient showing falls in FEV1 of 35% 20 min after exposure and of 30% 10 h after exposure compared with baseline. This response was not observed after inhalation of placebo (lactose) the day before the test (Fig. 1).

Taking into account the possible cross-reactivity between shrimp and other invertebrates, a blood determination of the tropomyosins Der p 10, Pen a 1 and Pen m 1 (Inmunocap^®^, Phadia AB, Uppsala, Sweden) was carried out, as well as the allergen Pen m 2 (family of Arginine kinase from *Penaeus monodon*) (ISAC^®^, Phadia AB, Uppsala, Sweden), which were negative. Only a positive value of specific IgE antibodies was obtained for Pen m 4, a SCBP, at a level of 6.7 ISU-E (Table 1).

**Table 1:** Allergens analyzed

	Allergen	Molecular weight (kDa)	IgE
Tropomyosin	Pen a 1	34–38	< 0.01 kU/L
	Pen m 1		< 0.3 ISU-E
	Der p10	32	< 0.01 kU/L
Arginine kinase	Pen m 2	40–45	< 0.3 ISU-E
SCBP	Pen m 4	20–25	6.7 ISU-E

*SCBP* sarcoplasmic calcium binding protein

It was concluded that the allergen Pen m 4, contained in the shrimp Gammarus powder, was the cause of the patient’s asthma. Other allergens such as myosin light chain, troponic C or triosephosphate isomerase and possible cross-reactions cannot be ruled out [6]. Since being diagnosed with OA due to Gammarus powder and receiving the recommendations to avoid exposure to this agent, the patient quit his job and remained asymptomatic.

Seafood sensitization occurs predominantly in the digestive tract but is also frequently caused by aerosol exposure during manipulation in factories or even in the domestic environment. The prevalence of OA in seafood workers is variable but there seems to be a strong correlation between the concentration of environmental allergens and sensitization to them [2, 9].

Shrimp proteins are high molecular weight agents which cause asthma by an immune mechanism mediated by IgE [2]. Several molecular allergens have been characterized; the tropomyosins Pen a 1, Pen m 1, Lit v 1 are the proteins most frequently involved in the sensitization of these patients and are responsible for a greater cross-reactivity with crustaceans, other arthropods and mollusks [6, 9].

Gammarus is a crustacean belonging to the genus of amphipods. It is not included in the group of common crustaceans that produce allergic reactions, such as *Palinurus* sp., *Penaeus* sp., *Leander* sp., *Homarus* sp., *Metapenaeus* sp. and *Pandalus* sp. [2]. This small shrimp is used in many European countries for the production of feed for turtles and domestic fish. In the last 20 years, five cases of sensitization to inhaled shrimp Gammarus have been published [2–5] in which rhinoconjunctivitis, urticaria and/or bronchial asthma were described.

Baur et al. [5] reported the case of a patient working in a fish food factory who developed allergic asthma due to shrimp Gammarus. Immunoblotting performed with the Gammarus extract showed several IgE-binding bands in the range of 14 to > 90 kDa. Gamboa et al. [3] published a case report of allergic rhinitis from sensitization to Gammarus in a patient using shrimp Gammarus as feed for a pet turtle. The immunoblotting showed IgE-binding to proteins of approximate molecular masses of 25, 32, and 40 kDa. IgE-binding to tropomyosin (35 kDa) was not observed. Finally, Fontan et al. [4] described three cases of allergy to Gammarus, two of them with cutaneous symptoms and one with OA. The patient’s sera showed different bands ranging from 100 to 20 kDa. In the three cases, very few or no cross-reactions with other common allergenic arthropods could be detected.

In the present case report, the clinical symptoms described by our patient with initial respiratory and subsequent alimentary symptoms indicate that the inhaled route was probably the primary pathway of sensitization. Moreover, the tests performed show undetectable IgE levels against tropomyosin (Pen a 1, Pen m 1). This result suggests that the sensitization to shrimp Gammarus in this patient occurred without the intervention of cross-reactivity with tropomiosin of other invertebrates, although cross-reactivity with SCBP of crayfish cannot be ruled out [7]. In fact, the patient was only sensitized to Pen m 1, a SCBP. SCBPs have a molecular weight of approximately 20 kDa and an isoelectric point of 5, and are stable to the action of heat [7–11]. Recent studies have pointed out the importance of SCBPs as shellfish allergens. Ayuso et al. [11] showed IgE recognition to these proteins in 85% of children allergic to shrimps. Moreover, SCBPs appear to be more potent basophil activators than tropomyosin, and so they have been associated with greater clinical reactivity to shrimp [11].

In conclusion, we present a case of a patient exposed to Gammarus powder who developed OA due to the exclusive sensitization to Pen m 4 allergen. The sensitization to Pen m 4 described here may identify a new allergen causing OA in these workers.

## Abbreviations

FEV1: forced expiratory volume in the first second
FVC: forced vital capacity
OA: occupational asthma
PBD: positive bronchodilator test
SCBP: sarcoplasmic calcium binding protein
